# Magnesium surface enrichment of CoFe_2_O_4_ magnetic nanoparticles immobilized with gold: reusable catalysts for green oxidation of benzyl alcohol†

**DOI:** 10.1039/c7ra13590d

**Published:** 2018-01-22

**Authors:** Wiury C. de Abreu, Marco A. S. Garcia, Sabrina Nicolodi, Carla V. R. de Moura, Edmilson M. de Moura

**Affiliations:** Departamento de Química, Universidade Federal do Piauí Teresina 64049-550 PI Brazil mmoura@ufpi.edu.br; Instituto Federal do Maranhão Buriticupu 65393-000 MA Brazil; Instituto de Física, Universidade Federal do Rio Grande do Sul Porto Alegre 91501-970 RS Brazil

## Abstract

Gold nanoparticles have shown excellent activity for selective oxidation of alcohols; such catalytic systems are highly dependent on the initial activation of the substrates, which must occur on the catalyst surface in heterogeneous catalysts. In many cases, an extra base addition is required, although the basicity of the support may also be of significant importance. Here, we explored the intrinsic basicity of magnesium-based enrichments on CoFe_2_O_4_ magnetic nanoparticles for the oxidation of benzyl alcohol using molecular oxygen as oxidant. The MgO and Mg(OH)_2_ enrichments enabled gold impregnation, which was not possible on the bare CoFe_2_O_4_ nanoparticles. The Au/MgO/CoFe_2_O_4_ and Au/Mg(OH)_2_/CoFe_2_O_4_ catalysts reached 42% and 18% conversion, respectively without base promotion, in 2.5 hour and 2 bar of O_2_. When the catalysts were tested with sub-stoichiometric amounts of base, they became more active (>70% of conversion) and stable in successive recycling experiments without metal leaching, under the same reaction conditions. We also showed the oxide phases of the enrichments performed using Rietveld refinements and how the Mg(OH)_2_ phase interferes with the activity of MgO-based materials.

## Abstract 2

Gold nanoparticles have shown excellent activity for selective oxidation of alcohols; such catalytic systems are highly dependent on the initial activation of the substrates, which must occur on the catalyst surface in heterogeneous catalysts.

## Graphical abstract

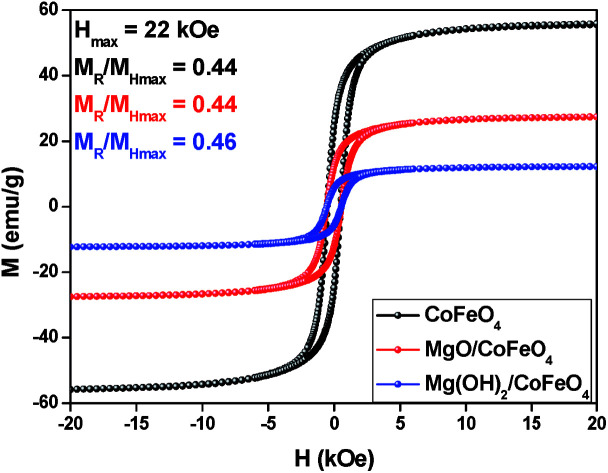

## 1. Introduction

The oxidation of alcohols to obtain aldehydes, ketones and carboxylic acids is essential for organic chemistry, since carbonyl compound derivatives are very important for academic and industrial research.^1^ Catalytic systems able to promote selective oxidations have gained significant attention in the last few years due to their vast potential. The proof lies in the fact that 50% of the published literature about gold nanoparticles (NPs) deals with oxidation reactions.^2^ Although numerous catalysts based mainly on palladium and platinum have been proposed,^3–7^ Au NPs have shown better selectivity than other metals in alcohol oxidations.^8–10^ Bi- and trimetallic systems may also be remarkably selective when compared to individual metals,^11–13^ however their synthesis usually demands more preparation steps and eventually they are more difficult to synthesize *via* traditional methods (*i.e.*, co-impregnation, co-precipitation).^14^ Systematic studies on the nature of the support, Au NPs size, catalyst preparation conditions and metal precursor have contributed to the design of more efficient catalysts.^15,16^ Some proposed insights deal with differences in reactivity considering the mechanism steps for oxidation of alcohols: metal alkoxide formation, β-elimination and metal-hydride formation, with a subsequent catalyst regeneration.^17,18^ In aqueous-phase, a basic solution induces the initial deprotonation of the alcohol to form an alkoxy intermediate.^19,20^ In solventless conditions, the support and/or the base have to act on the activation of the alcohol on the catalyst surface. The principal roles of the catalyst support are ascribed to stabilization of the active components, metal dispersion and surface-area increasing,^21^ carrier effects,^22^ roughness and intrinsic basicity,^23,24^ the latter being highly interesting for alcohols oxidation. Charge-density models predict the strength of oxygen Lewis basic sites on alkaline earth metal oxides is O_2_ > OH > H_2_O > H_3_O^+^.^25^ This trend is important since the choice for a catalytic support may influence its activity and selectivity. Magnesium oxide and magnesium hydroxide are examples of important supports used for alcohol oxidation without base addition.^23,24,26^ However, there is a lack of studies that perform a real comparison between them, with or without extra base addition, since the basicity may not be the prominent effect. Also, the association of oxides and magnetic NPs allows the preparation of multifunctional nanomaterials that may be easily collected from the reaction medium upon external magnetization.^27–31^ We have published a mixed magnetic Au/MgO/MgFe_2_O_4_ catalyst very active and recyclable for benzyl alcohol oxidation that was able to react with or without extra base addition, and we discussed the important role the base had on its stability.^32^ New possibilities of catalyst syntheses with different magnetic supports are interesting and allow evaluating specifically the enrichments performed, even when the cations used for the magnetic support production present no basicity. Cobalt ferrite (CoFe_2_O_4_) NPs are somehow not considered for gold-catalyzed alcohol oxidation reactions,^33^ although its remarkable stability in air up to 1000 °C, which may not be the case for Fe_3_O_4_ NPs, unless some capping procedure is performed.^34^

Carrying on our prior studies in the field of magnetic separation and gold-based catalysts for solventless benzyl alcohol oxidation,^32^ CoFe_2_O_4_ spinel-type supports with different magnesium surface enrichments were investigated to comprehend how the surface basicity influences catalytic reactions. Our aim herein was to study the effect on the catalysis of MgO and Mg(OH)_2_ enrichments using molecular oxygen as oxidant and their induction outcome on the oxidation, with or without extra base addition. We have also studied the recycling potential of the catalysts prepared.

## 2. Experimental section

### 2.1 CoFe_2_O_4_ preparation

Cobalt ferrite NPs were prepared by a co-precipitation method.^35^ In a typical procedure, two aqueous solutions of CoCl_2_·6H_2_O (2.5 mL, 4.1 mmol, diluted in an aqueous solution of 2 mol L^−1^ of HCl) and FeCl_3_·6H_2_O (5 mL, 8.2 mmol) were mixed and added to 125 mL of a ammonium hydroxide solution (0.7 mol L^−1^) under vigorous stirring (1000 rpm, Arec X, Velp Scientifica). A black precipitate immediately was formed. After a 2 hour stirring, the product was collected with a permanent magnet (neodymium magnet) and the supernatant removed. The solid was washed three times with hot water (200 mL, 80 °C) and once with acetone (100 mL) before drying in an oven at 80 °C. Then, the material was calcined in a muffle furnace in air at 800 °C for 3 hours, at a heating rate of 10 °C min^−1^.

### 2.2 MgO/CoFe_2_O_4_ and Mg(OH)_2_/CoFe_2_O_4_ preparation

The CoFe_2_O_4_ enrichments with MgO and Mg(OH)_2_ (both commercially acquired) were performed by a impregnation method.^32^ CoFe_2_O_4_ and Mg(OH)_2_ were mixed in a mass ratio of 1 : 5 in water under stirring (1000 rpm) for 24 hours. Then, the material was dried in an oven at 100 °C for 12 hours. The same procedure was performed for MgO enrichment, however, instead of water, acetone was used in the procedure.

### 2.3 Preparation of Au/MgO/CoFe_2_O_4_ and Au/Mg(OH)_2_/CoFe_2_O_4_ NPs

The gold-supported catalysts were synthesized using a modified sol-immobilization method described elsewhere.^36^ For the syntheses, 1.80 mL of an aqueous solution of 2.0 wt% polyvinyl alcohol (PVA, 36 mg) was added under vigorous stirring to an aqueous solution of HAuCl_4_ (172.5 mg, 300 mL). After a dropwise addition of 7.65 mL of NaBH_4_ (0.1 mol L^−1^), the metal reduction was achieved, transforming the solution into a dark purple color. After that, the solution was stirred extra 30 minutes. Then, 1.5 g of one of the supports was added to the sol and stirred for three hours at room temperature. The solid was magnetically separated with a permanent magnet and washed three times with hot water (200 mL, 80 °C) and once with acetone (100 mL) before drying in an oven at 80 °C for 12 hours.

### 2.4 Catalytic reactions

Oxidation reactions were performed in a 100 mL Fischer–Porter glass reactor at 2 bar of O_2_ and 100 °C. In a typical reaction, the reactor was loaded with the catalyst (4.1 mmol of Au) and benzyl alcohol (9.6 mmol) under magnetic stirring. The temperature and stirring were maintained by a stirring plate connected to a digital controller (Arec X, Velp Scientifica). Usually, the reaction time was 2.5 h, except when mentioned. At the end of reaction, the catalyst was recovered by placing a permanent magnet on the reactor wall. The products were analyzed by gas chromatography (GC) using *p*-xylene as standard. The catalyst was used several times and washed with CH_2_Cl_2_ before each recycling.

### 2.5 Characterizations

Transmission electron microscopy (TEM) images were obtained using JEOL JEM 2100 (operating at 110 kV) and Tecnai G2 (operating at 200 kV) microscopes. Samples for TEM were prepared by drop casting an isopropanol suspension of the samples over a carbon-coated copper grid, followed by drying under ambient conditions. Furnace Atomic Absorption Spectroscopy (FAAS) was performed using a Shimadzu AA-6300. BET and BJH surface areas and pore size distribution on the materials were obtained on a Quantachrome Novawin equipment by N_2_ physisorption at 77 K. The magnetic characterizations were performed by using an EZ9 MicroSense vibrating sample magnetometer (VSM) at room temperature with a magnetic field cycled between −22 kOe and +22 kOe. Thermogravimetric (TG) measurements were performed on a DTG-60/DTG-60A SHIMADZU equipment (TG/DTA simultaneous measuring instrument). The experiments were conducted in the temperature range of 30 to 1100 °C using Pt crucible with approximately 15 mg of sample, heating rate of 10 °C min^−1^, under dynamic nitrogen atmosphere (50 mL min^−1^). The equipment conditions were verified with a standard reference of CaC_2_O_4_·H_2_O. The blank TG/DTG curves were obtained under the same experimental conditions for baseline correction. The X-ray diffractograms (DRX) were obtained using a Bruker D8 Advance equipment using monochromatic Cu Kα radiation (*λ* = 1.54056 Å) and graphite monochromator. The voltage of the copper emission tube was 40 kV and the filament current was 40 mA, at a 2*θ* range from 10° to 90° with a 0.02° step size and measuring time of 5 s per step. The X-ray photoemission spectra was obtained with a Scienta Omicron ESCA + spectrometer system equipped with an EA 125 hemispherical analyzer and a XM 1000 monochromated X-ray source in Al kα (1486.7 eV). The X-ray source was used with a power of 280 W as the spectrometer worked in a constant pass energy mode of 50 eV. The phases composition identification was performed by Rietveld refinement using GSAS EXPGUI 2012 software.

## 3. Results and discussion

**Fig. 1:**
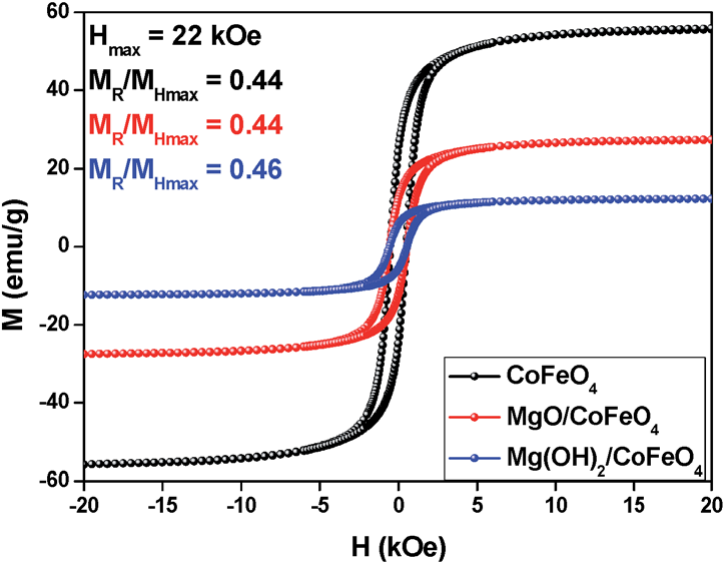
Magnetization as a function of the applied magnetic field for bare CoFe_2_O_4_, MgO/CoFe_2_O_4_, Mg(OH)_2_/CoFe_2_O_4_ at room temperature. The hysteresis loops were recorded with a magnetic field cycled between −22 kOe and +22 kOe.

Magnetic separation has arisen as a highly efficient catalyst separation toll compared to other separation unit operations. A co-precipitation method using Fe^3+^ and Co^2+^ under alkaline conditions was the choice for the synthesis of the magnetic particles studied herein. This simple and eco-friendly procedure is the most widely used for CoFe_2_O_4_ NPs synthesis, although polydisperse NPs are obtained. Generally, however, the final material has satisfactory magnetic proprieties.^37^ Inorganic oxides, such the ones used in the studies performed – MgO and Mg(OH)_2_ – are extensively used in catalysis and may be associated to magnetic NPs. Some of us have published that MgFe_2_O_4_ with or without MgO modification was efficiently impregnated with PVA-stabilized Au NPs, enabling catalytic activities studies for both systems.^32^ Nevertheless, Au NPs uptake over CoFe_2_O_4_ NPs was only possible with the oxide/hydroxide enrichments. Therefore, after the CoFe_2_O_4_ amendments, gold NPs were immobilized by a wet-impregnation method using PVA-stabilized Au NPs. To guarantee the impregnation or total covering of the CoFe_2_O_4_ with the oxides, as well as the gold immobilization, has little or no impact on the magnetic property of NPs, magnetization as a function of the applied magnetic field, *M*(H), measurements were performed (Fig. 1). The materials' characteristic hysteresis loops were obtained for applied magnetic field ranging from +22 kOe to −22 KOe. As a first sight, one may notice the CoFe_2_O_4_ NPs exhibit hysteresis loops with similar shape in the three measurements performed (for bare CoFe_2_O_4_, MgO/CoFe_2_O_4_, Mg(OH)_2_/CoFe_2_O_4_). This is better evidenced by comparing the normalized curves (not shown). The sample CoFe_2_O_4_ impregnated with MgO presented a saturation magnetization (under the maximum magnetic field used for the measurements) of 27.5 emu g^−1^ and the solid loaded with Mg(OH)_2_ presented 12.2 emu g^−1^. Although these values are smaller than the observed for the bare CoFe_2_O_4_ (55.7 emu g^−1^), the solids can still be efficiently separated from the reaction medium upon magnetization with a permanent Nd_2_F_14_B magnet, as observed in the recycling experiments. The magnetic response of both enriched supports is basically the same and matches to the response observed for the bare NPs, corroborating the information obtained before that attested the materials are easily recovered once necessary. The catalysts preparation procedures affected the maximum magnetic signal (*M*_Hmax_) when compared to support without modification: MgO/CoFe_2_O_4_ (*M*_Hmax_ ≈ 27.2 emu g^−1^) and Mg(OH)_2_/CoFe_2_O_4_ (*M*_Hmax_ ≈ 12.57 emu g^−1^) decreased twice and five times, respectively, when comparting them to bare NPs (56.90 emu g^−1^). The reductions are expected since the molar ratio oxide/CoFe_2_O_4_ was 5 for both samples. Once again, the proof that the magnetic properties are not affected by the use of the oxides can be seen by the remnant magnetization (*M*_R_/*M*_Hmax_), which was practically the same for the three cases. The field coercivity slightly change with the immobilization procedures carried out: 510, 564 and 560 Oe for CoFe_2_O_4_, MgO/CoFe_2_O_4_, Mg(OH)_2_/CoFe_2_O_4_ respectively.

**Fig. 2:**
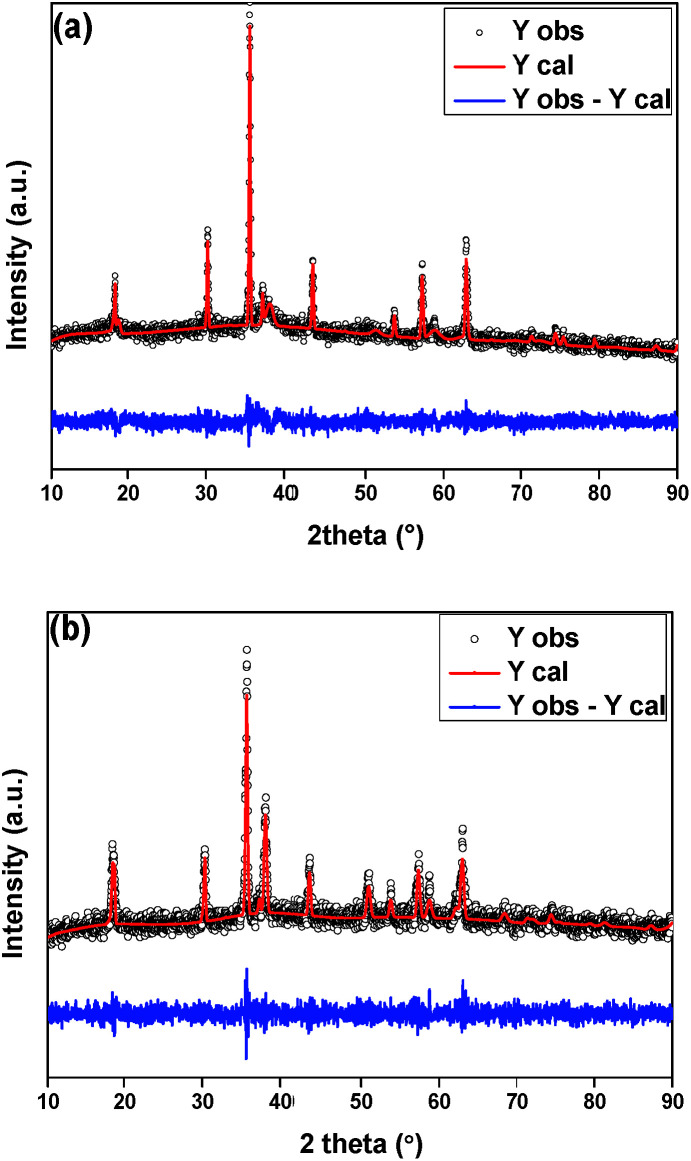
Rietveld refinement plot for (a) Au/MgO/CoF_2_O_4_ and (b) Au/Mg(OH)_2_/CoF_2_O_4_ catalysts, showing the observed, calculated and difference pattern.

**Fig. 3:**
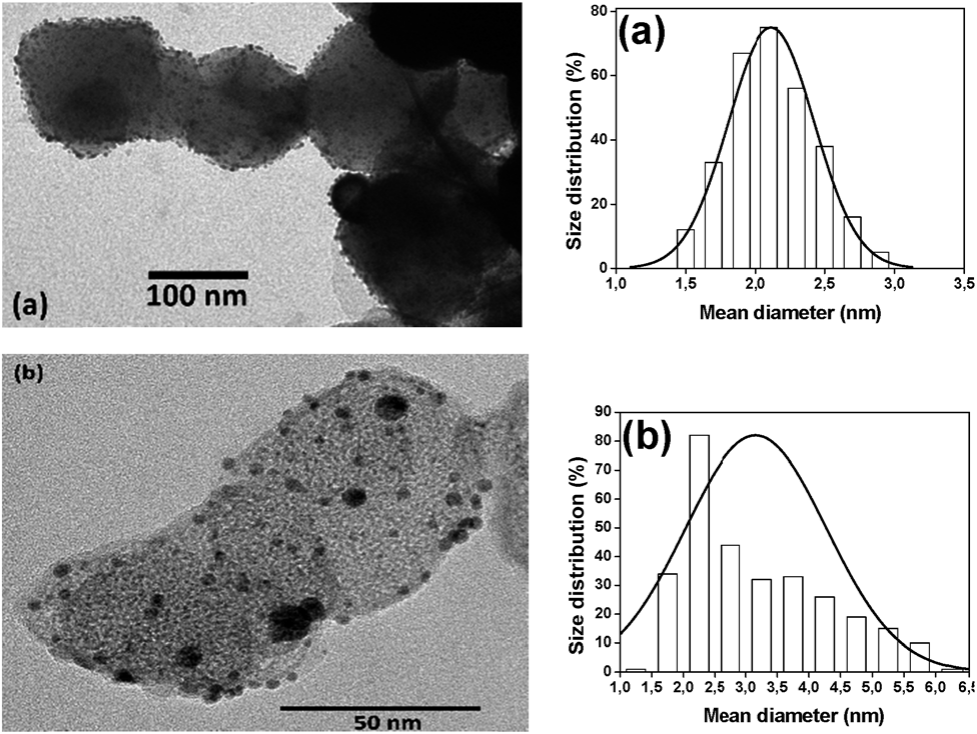
TEM images of (a) Au/MgO/CoF_2_O_4_ and (b) Au/Mg(OH)_2_/CoF_2_O_4_ catalysts and the corresponding size distribution histograms.

Since the synthesis procedures use water for the Au NPs impregnation, it's essential to quantify the amount of crystalline phases of the magnesium-based materials impregnated on the CoFe_2_O_4_ NPs. It's known that MgO may be converted to Mg(OH)_2_ in presence of water,^38^ hence any catalytic singularity may be better explained possessing such data. The phase composition of the prepared supports was obtained by Rietveld refinement. Fig. 2a shows the XRD pattern of the sample Au/MgO/CoF_2_O_4_. The diffraction pattern indexed three crystalline phases: CoFe_2_O_4_ (ICSD 109045), MgO (ICSD 31051) and Mg(OH)_2_ (ICSD 34401). Data obtained resulted in a majority phase comprised of cubic MgO (61%). The remained phases were trigonal Mg(OH)_2_ (33%) and cubic CoFe_2_O_4_ (6%). For the sample Au/Mg(OH)_2_/CoF_2_O_4_ (Fig. 2b), the main phase was cubic Mg(OH)_2_ (92%) and the remaining phase was of trigonal CoFe_2_O_4_ (8%). For both samples, due to the small amount of gold on the support, no Au phase was observed. The low concentration of Au NPs (2 wt% for both catalysts, determined by FAAS, Table 2) also hampered the size definition by XRD; however TEM analyses clearly showed well-dispersed Au NPs onto both supports (Fig. 3). Basically, both catalysts present the same NPs size and very narrow size distribution, most likely because the syntheses use an efficient pre-formed colloidal approach. The mean diameter for Au/MgO/CoFe_2_O_4_ catalyst was 2.09 ± 0.01 nm and for the Au/Mg(OH)_2_/CoFe_2_O_4_ catalyst was 2.31 ± 0.09 nm.

**Fig. 4:**
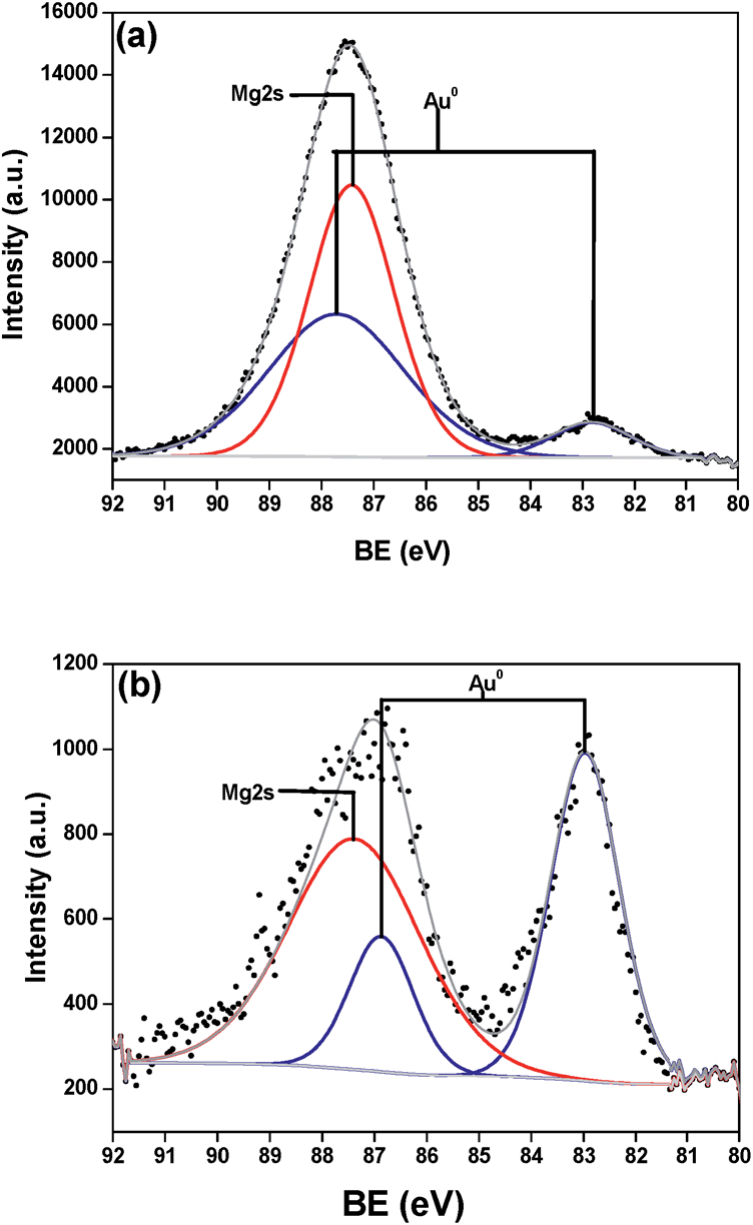
XPS spectra of samples (a) Au/MgO/CoF_2_O_4_ and (b) Au/Mg(OH)_2_/CoF_2_O_4_. Mg 2s is represented by the red line fitting and the Au 4f fitting is represented by the blue line.

Although information related to magnesium enrichments were performed by Rietveld refinement, gold was not observable by the technique. Hence, the surface chemical composition of the as-prepared catalysts was analyzed by XPS with a focus on Au species chemical states. Fig. 4 presents the spectra for Mg 2s and Au 4f. There is an overlapping of gold peaks with the high intensity Mg peaks; however the deconvolution procedure performed showed two doublets spin–orbit components of Au 4f, separated by 4.33 eV (Au/MgO/CoF_2_O_4_, Fig. 4a) and 3.70 eV (Au/Mg(OH)_2_/CoF_2_O_4_, Fig. 4b). These two states (Au 4f_7/2_ and Au 4f_5/2_) are consistent to Au(0) species.^39^ The deconvoluted states did not present residual amounts of Au(i), but the cations cannot be completely excluded. The peaks intensity variances of Au and Mg species suggest the gold content is different in the samples, nevertheless FAAS analysis confirmed 2 wt% for both catalysts. The difference may be explained by some sort of agglomeration of Au NPs on the analyzed area.

The Au/MgO/CoFe_2_O_4_ and Au/Mg(OH)_2_/CoFe_2_O_4_ catalysts were applied in the solventless oxidation of benzyl alcohol without additional base. The experiments were performed at 100 °C, since TG analyses showed the catalysts are stable under this temperature (ESI, Fig. S1 and S2†). The results are presented in Table 1. Both supports used for the studies presented no activity for the proposed reaction (entries 1 and 2). However, the immobilization of the Au NPs on them provided catalysts able to oxidase benzyl alcohol without additional base. Basically, the intrinsic basicity of the materials was sufficient to promote such reactions at 2 bar of O_2_. Predictably, Au/MgO/CoFe_2_O_4_ catalyst (entry 3) was more active than the Au/Mg(OH)_2_/CoFe_2_O_4_ catalyst (entry 4), since the former has majority phase comprised of MgO, which presents a basicity strength higher than the other material, with 98% of Mg(OH)_2_.^25^ Besides that, MgO surface has a much higher capacity of adsorbing and activating the substrate.^40^ The selectivity are also in accordance to expected, since the lower the basicity, the lower the tendency to form benzoic acid.^12^ Therefore, the Au/Mg(OH)_2_/CoFe_2_O_4_ has a higher selectivity for benzaldehyde than the Au/MgO/CoFe_2_O_4_ catalyst. Costa *et al.* performed benzyl alcohol oxidation with Au/MgO catalyst, under more severe conditions, and obtained just 15% in 4 hours, attesting the higher activity of the Au/MgO/CoFe_2_O_4_ catalyst.^23^ Increasing the reaction pressure from 2 to 4 bar of O_2_ had no effect on the catalytic activity. Decreasing to 1 bar, the catalysts achieved similar activities, with no significant selectivity modification.

**Table 1:** Oxidation reactions of benzyl alcohol without base addition^a^

Entry	Catalyst	Conversion (%)	Selectivity (%)	
			Benzaldehyde	Benzoic acid
1	MgO/CoFe_2_O_4_	0	—	—
2	Mg(OH)_2_/CoFe_2_O_4_	0	—	—
3	Au/MgO/CoFe_2_O_4_	42	62	38
4	Au/Mg(OH)_2_/CoFe_2_O_4_	18	73	27

a Reaction conditions (solventless): 9.6 mmol of benzyl alcohol, 4.1. mmol of Au (catalyst), 2 bar of O_2_, 2.5 h.

The lower activity of the Au/Mg(OH)_2_/CoFe_2_O_4_ catalyst may be also associated to its porosity and surface area. For such conclusions, textural characteristics of both catalysts were measured by N_2_ physisorption technique (ESI, Fig. S3 and S4†). The catalysts Au/MgO/CoFe_2_O_4_ (isotherm type IV) and Au/Mg(OH)_2_/CoFe_2_O_4_ (isotherm type III) presented values of specific surface are of 102 and 44 m^2^ g^−1^, respectively (Table 2). The pore diameter and total pore volume of the former material are also higher than the observed for the supported enriched with Mg(OH)_2_. These values are in accordance to literature^41^ and considering the catalyst dispersion are similar, as seen in Fig. 3, the lower values of the latter catalyst, associated to its basicity, may explain just 18% of conversion.

**Table 2:** Chemical analysis and surface proprieties measured by N_2_ physisorption of the catalysts

Catalyst	Au content (%)	Surface area (m^2^ g^−1^)	Pore diameter (Å)	Total pore volume (cm^3^ g^−1^)
Au/MgO/CoFe_2_O_4_	2.0	102	83	0.46
Au/Mg(OH)_2_/CoFe_2_O_4_	2.0	44	28	0.28

Further studies on the oxidation of benzyl alcohol were conducted using bases as reaction promoters (Table 3) in sub-stoichiometric conditions. For the Au/MgO/CoFe_2_O_4_ catalyst, base addition promoted an augmentation on the catalytic activity; however, no prominent effect on the conversion was observed when KOH was used (entry 5). In 2.5 h, 50% of conversion was obtained, while the base-free experiment reached 42% of alcohol transformation. The selectivity to benzoic acid was higher for the KOH addition experiment, as expected, since the base would induce the complete oxidation of the substrate. The same selectivity effect was observed for the Au/Mg(OH)_2_/CoFe_2_O_4_ material, *i.e.*, higher production of benzoic acid (entry 6) when compared to the base-free condition reactions (entry 4). Nevertheless, one may observe a catalyst activity increasing. Without base, only 18% of conversion was obtained; KOH addition made the reaction advance to 54% of conversion. The K_2_CO_3_ addition promoted noticeable conversion increasing for both catalysts (entries 7 and 8), being more active in the Au/MgO/CoFe_2_O_4_ system. The conversions for Au/MgO/CoFe_2_O_4_ and Au/Mg(OH)_2_/CoFe_2_O_4_ were 86% and 77%, respectively. The selectivity for benzoic acid was the highest achieved and is quite similar for the materials. In an attempt to study the Mg(OH)_2_ influence on the Au/MgO/CoFe_2_O_4_ catalyst, a reaction was performed with this base addition in the same amount of the other promoters before (entry 9). The catalyst presented similar results to the observed without base addition (entry 3). The reason for this may be the strong basicity of the MgO compared to Mg(OH)_2_, which have shown a lower activity promotion before, when used as catalyst enrichment. Ferraz *et al.*^20^ rationalized that in non-aqueous phases, the support or reaction promoter must act on the catalyst surface, different from aqueous basic solution, which favor the initial deprotonation of the alcohol. Thus, the most efficient interaction with the surface was performed by the K_2_CO_3_, which associated to the intrinsic higher basicity of MgO (on Au/MgO/CoFe_2_O_4_) reached the best activity under the chosen reaction conditions.

**Table 3:** Oxidation reaction of benzyl alcohol with base addition using Au/MgO/CoFe_2_O_4_ and Au/Mg(OH)_2_/CoFe_2_O_4_ catalysts^a^

Entry	Catalyst	Base added	Conversion (%)	Selectivity (%)	
				Benzaldehyde	Benzoic acid
5	Au/MgO/CoFe_2_O_4_	KOH	50	47	53
6	Au/Mg(OH)_2_/CoFe_2_O_4_	KOH	54	52	48
7	Au/MgO/CoFe_2_O_4_	K_2_CO_3_	86	19	81
8	Au/Mg(OH)_2_/CoFe_2_O_4_	K_2_CO_3_	77	24	76
9	Au/MgO/CoFe_2_O_4_	Mg(OH)_2_	46	78	22

a Reaction conditions (solventless): 9.6 mmol of benzyl alcohol, 4.1 mmol of Au (catalyst), 0.33 mmol of base, 2 bar of O_2_, 2.5 h.

**Fig. 5:**
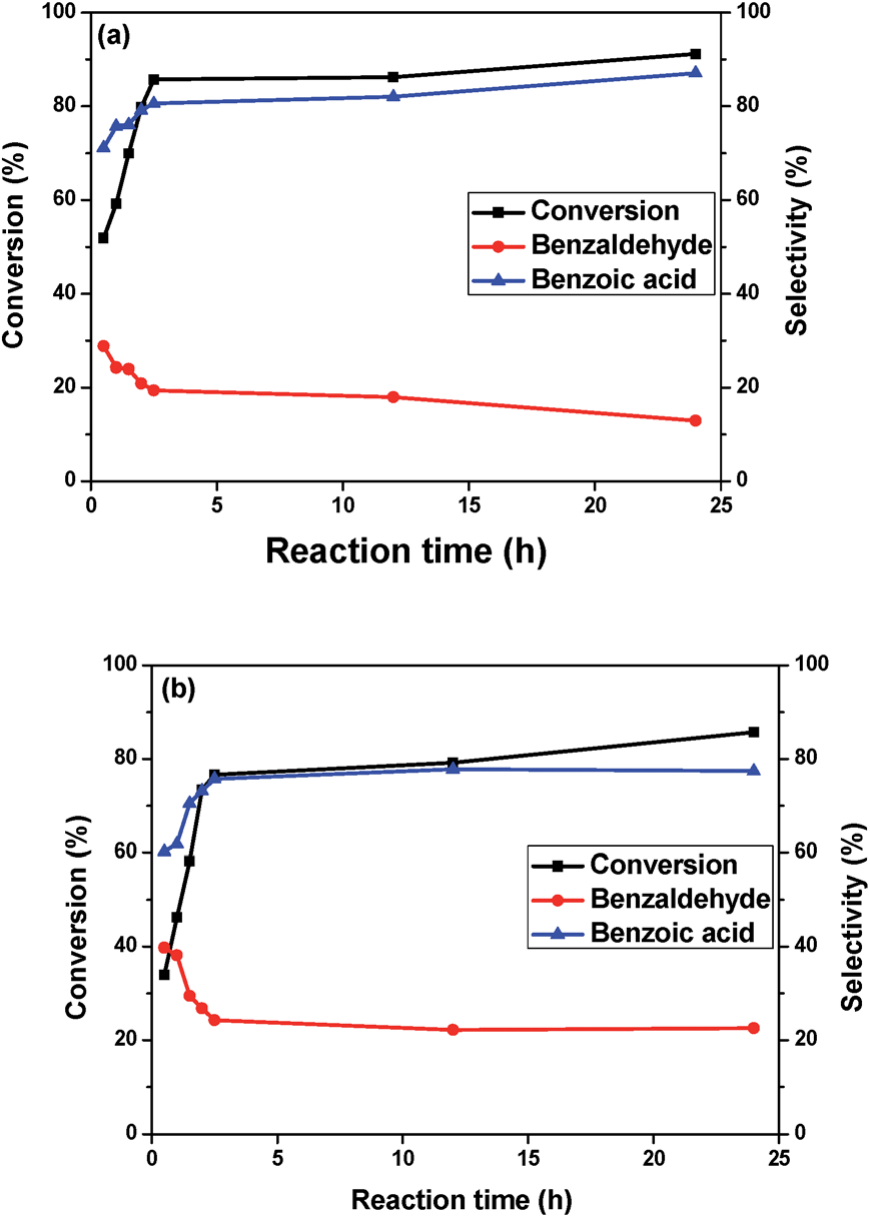
Influence of the reaction time on the conversion and selectivity in the oxidation of benzyl alcohol over (a) Au/MgO/CoFe_2_O_4_ and (b) Au/Mg(OH)_2_/CoFe_2_O_4_ catalysts.

The conversion *vs.* time profile of the reaction performed with the Au/MgO/CoFe_2_O_4_ catalyst promoted by K_2_CO_3_ is presented in Fig. 5a. The benzyl alcohol conversion was monitored over a 24 hour period. The conversion increasing is more pronounced in the first 2.5 hours of reaction and became moderate up to 24 hours. Basically, after 2.5 hours, the conversion slightly changed; in 12 hours, the conversion augmentation observed was of just 2%. In 24 hours, the conversion increasing was of 5%. The same profile was observed for the Au/Mg(OH)_2_/CoFe_2_O_4_ catalyst (Fig. 5b), varying evidently, the conversions obtained. The selectivity for both systems were essentially the same after the quasi-plateau of activity observed.

**Fig. 6:**
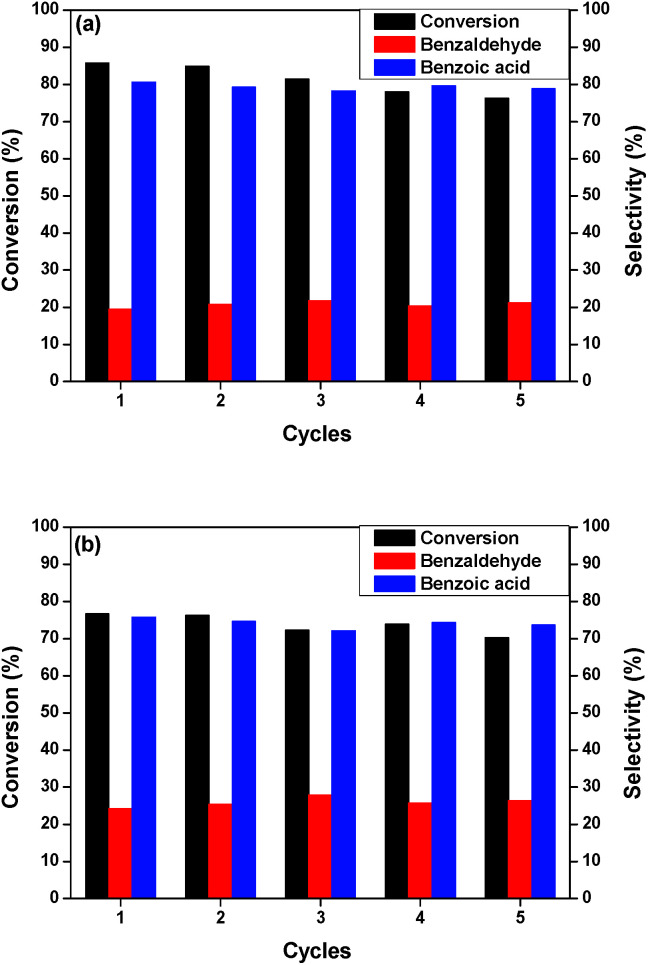
Recycling of (a) Au/MgO/CoFe_2_O_4_ and (b) Au/Mg(OH)_2_/CoFe_2_O_4_ catalysts in the oxidation of benzyl alcohol.

The stability of the catalysts was evaluated using the same reaction conditions and K_2_CO_3_ as base promoter. The catalysts were assessed in five successive reactions. After each reaction, the catalyst was washed with water to remove the base from the previous reaction and with CH_2_Cl_2_. The same quantity of base was added for each cycle. As seem in Fig. 6, both catalysts presented a high stability on the recycling tests, maintaining their selectivity. The Au/MgO/CoFe_2_O_4_ catalyst presented a minor drop in its conversion – less than 10% – showing the possibility of being still used several times. The Au/Mg(OH)_2_/CoFe_2_O_4_ catalyst exhibited a catalytic activity drop close to 6%. No Au leaching was observed in both systems after each run, as attested by FAAS. Considering the stability of the proposed systems and their easy syntheses, the utilization of just 3.4 mol% of base is an advantage; even with some low deactivations, as stated. Estrada *et al.*^40^ reported Au NPs supported on MgO are *ca.* 50% more active in benzyl alcohol oxidation than Mg(OH)_2_, however with such a small quantity of base we were able to circumvent this drawback.

## 4. Conclusion

Heterogeneous magnesium-based gold catalysts were effective for aerobic oxidation of benzyl alcohol. The CoFe_2_O_4_ magnetic support presented a high magnetization, which met our expectations for catalyst separation from the reaction medium. The MgO or Mg(OH)_2_ enrichments performed on the CoFe_2_O_4_ NPs were essential for the catalyst synthesis, since without its modification, no Au NPs impregnation was possible. In addition, the intrinsic basicity of the magnesium compounds allowed the reaction to proceed without base promoter. The Au/MgO/CoFe_2_O_4_ and Au/Mg(OH)_2_/CoFe_2_O_4_ catalysts reached 42% and 18% of conversion, respectively, without base promotion, in 2.5 hour and 2 bar of O_2_, showing the differences on the basicity between the oxide and hydroxide. The addition of a sub-stoichiometric amount of K_2_CO_3_ was found to be the best condition for the oxidation, improving the catalytic activity further to yields close to 80% or higher. The selectivity changed towards acid production (>70%) and the catalysts were able to react in successive cycles without Au leaching and a significant loss of activity. For the systems developed, with base addition, the choice between MgO or Mg(OH)_2_, although presenting some difference of the activity, were not that expressive.

## Conflicts of interest

There are no conflicts to declare.

## Supplementary Material

RA-008-C7RA13590D-s001
