# Supplementary material for: Magnesium surface enrichment of CoFe2O4 magnetic nanoparticles immobilized with gold: reusable catalysts for green oxidation of benzyl alcohol
Source: RSC Adv. 2018 Jan 22;8(7):3903–9. doi: 10.1039/c7ra13590d (PMC9077762; doi:10.1039/c7ra13590d)
Supplement: RA-008-C7RA13590D-s001 [file RA-008-C7RA13590D-s001.pdf]

Electronic Supplementary Material (ESI) for RSC Advances.
This journal is © The Royal Society of Chemistry 2018

Electronic Supplementary Information

Magnesium surface enrichment of $CoFe_2O_4$ magnetic nanoparticles immobilized with gold: reusable catalysts for green oxidation of benzyl alcohol

Wiury C. de Abreu,[ab] Marco A. S. Garcia,[a] Sabrina Nicolodi,[c] Carla V. R. de Moura,[a] and Edmilson M. de Moura*[a]

a. Departamento de Química, Universidade Federal do Piauí, Teresina 64049-550, PI, Brazil.

b. Instituto Federal do Maranhão, Buriticupu 65393-000, MA, Brazil.

c. Instituto de Física, Universidade Federal do Rio Grande do Sul, Porto Alegre 91501-970, RS, Brazil.

* E-mail Correspondence: mmoura@ufpi.edu.br

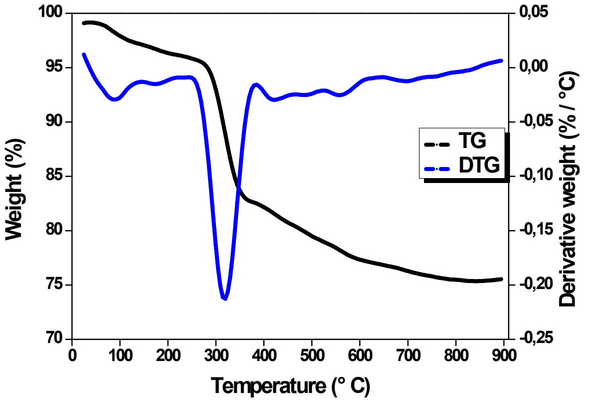

Figure S1 TG and DTG of Au/MgO/$CoFe_2O_4$ catalyst.

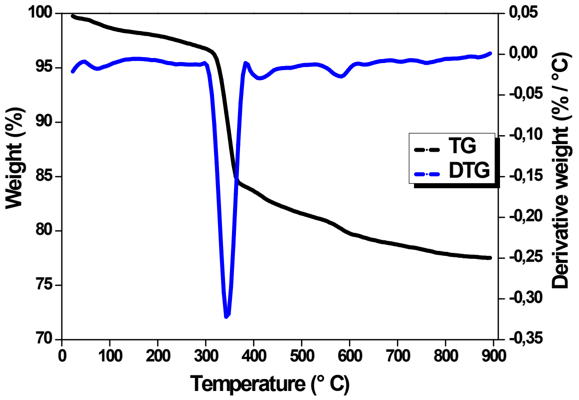

Figure S2 TG and DTG of Au/$Mg(OH)_2$/$CoFe_2O_4$ catalyst.

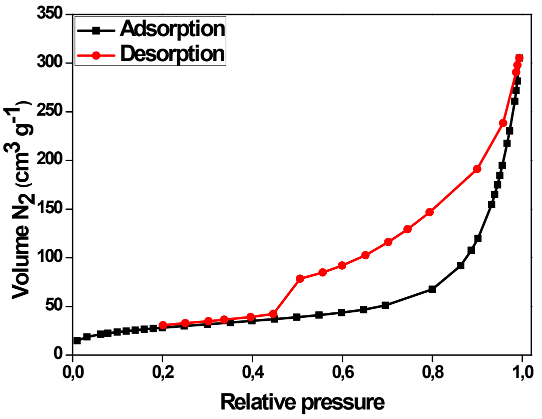

Figure S3 N_2 adsorption/desorption isotherms (at room temperature) for Au/MgO/$CoFe_2O_4$.

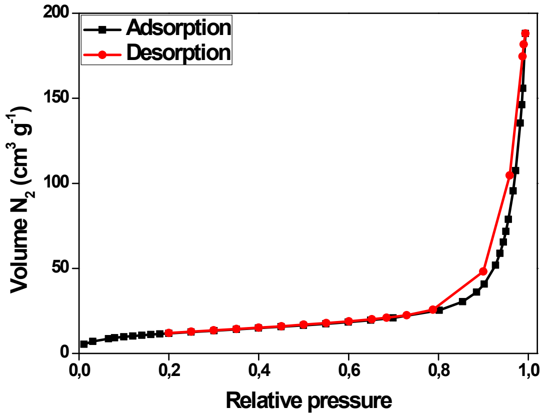

Figure S4 N_2 adsorption/desorption isotherms (at room temperature) for Au/$Mg(OH)_2$/$CoFe_2O_4$.
